# Prospective evaluation of occlusion time and force distribution in implant-supported fixed prostheses using the T-scan

**DOI:** 10.3389/fdmed.2026.1747002

**Published:** 2026-05-12

**Authors:** Soumya Raj, Manoj Shetty, Chethan Hegde

**Affiliations:** 1 Department of Prosthodontics & Crown and Bridge, AB Shetty Memorial Institute of Dental Sciences, NITTE (Deemed to be University), Mangaluru, Karnataka, India; 2 Department of Oral Implantology, AB Shetty Memorial Institute of Dental Sciences, NITTE (Deemed to be University), Mangaluru, Karnataka, India

**Keywords:** implant-supported prosthesis, occlusion, occlusion time, relative occlusal forces, T-Scan

## Abstract

**Background:**

Osseointegrated implants lack a periodontal ligament, resulting in altered occlusal force distribution and reduced proprioception. Implant-Protected Occlusion (IPO) has been advocated to minimize mechanical stress through optimized load distribution. Nevertheless, the long-term stability of occlusal contacts and force distribution in multiple implant-supported prostheses remains unclear. This study evaluates changes in relative occlusal force (ROF) and occlusion time (OT) following placement of posterior multiple implant-supported prostheses and their effect on force equilibration within the arch

**Methods:**

A prospective follow-up study was conducted on 25 patients rehabilitated with posterior multiple implant-supported fixed prostheses. The T-Scan III system was used to quantify relative occlusal force (ROF) and occlusion time (OT) at baseline, immediately after prosthesis placement, and at 3- and 6-month follow-ups. The contralateral side served as control. Occlusal variations over time were analyzed using Wilcoxon signed-rank and Friedman tests.

**Results:**

A significant increase in ROF for implant-supported prostheses was observed over time, with force distribution achieving greater equilibration by 6 months. Initially, higher forces were recorded in the anterior segments, which progressively shifted posteriorly to restore balance. Occlusion time also increased gradually, aligning with natural dentition.

**Conclusions:**

Occlusal parameters in implant-supported prostheses are dynamic and subject to change over time. Periodic monitoring and adjustment are essential to prevent excessive loading and to maintain functional harmony.

## Introduction

Implant-supported prostheses have emerged as a contemporary and predictable treatment option for the rehabilitation of edentulous spaces, restoring masticatory efficiency, phonetics, esthetics, and overall oral function, thereby improving patient quality of life (1). Despite their clinical success, biomechanical differences between implants and natural teeth necessitate careful occlusal consideration.

Unlike natural teeth, osseointegrated implants lack a periodontal ligament (PDL), resulting in diminished proprioception, reduced axial mobility, and altered load distribution (2). Natural teeth demonstrate physiologic mobility mediated by the PDL, allowing minor axial and rotational displacement that buffers occlusal forces. In contrast, implants form a rigid ankylotic interface with bone, permitting only minimal displacement determined by the viscoelastic properties of bone. Consequently, occlusal forces applied to implant-supported prostheses are transmitted more directly to the peri-implant bone, increasing susceptibility to mechanical complications such as screw loosening, component fracture, crestal bone loss, and implant failure (2).

To mitigate these risks, conventional occlusal principles are modified in implant dentistry. Implant-Protected Occlusion (IPO) has been advocated as a strategic approach to reduce mechanical stress by controlling the magnitude, direction, duration, and distribution of occlusal forces (3). In clinical practice, this often translates to lighter or slightly delayed occlusal contacts on implant restorations relative to natural teeth.

However, the long-term stability of occlusal contacts achieved through IPO remains uncertain. Occlusal forces are dynamic and influenced by magnitude, direction, duration, and timing. Over time, interocclusal friction may lead to enamel wear and altered occlusal morphology, thereby modifying contact patterns (4–6). In natural dentition, adaptive mechanisms such as mesial drift, compensatory eruption, and temporomandibular joint remodeling facilitate redistribution of occlusal loads and maintenance of functional harmony (5–7). Implants, however, lack PDL-mediated proprioception and adaptive mobility, limiting their capacity to accommodate to these changes. This biomechanical discrepancy becomes particularly relevant when implants oppose natural teeth, where progressive occlusal changes may predispose the prosthesis to overload (8).

Occlusal adaptation is not solely dependent on eruptive changes of antagonistic teeth but also influenced by neuromuscular coordination within the stomatognathic system. Alterations in masticatory muscle activity and mandibular posture may contribute to redistribution of occlusal contacts over time (5, 6). Understanding how implant-supported prostheses respond to this dynamic occlusal environment is essential for enhancing long-term functional stability.

Occlusion may be assessed using qualitative or quantitative methods. Articulating paper provides visual assessment but lacks force quantification (9). The T-Scan system (Tekscan Inc.) offers real-time digital analysis of relative occlusal force (ROF), contact timing, and force distribution, enabling objective evaluation of occlusal dynamics in both natural dentition and implant prostheses (10, 11).

Previous studies have primarily evaluated single posterior implant restorations and reported temporal changes in force distribution and occlusion time (2, 7). However, prospective longitudinal data examining posterior multiple implant-supported fixed prostheses remain limited. Whether multiple implants exhibit similar adaptive equilibration within the dental arch has not been sufficiently explored.

Therefore, the primary objective of this prospective study was to evaluate changes in relative occlusal force (ROF) and occlusion time (OT) in posterior multiple implant-supported fixed prostheses over a 6-month period using digital occlusal analysis. A secondary objective was to assess whether these prostheses demonstrate progressive equilibration toward natural dentition within the arch.

## Materials and methods

The data for this *in vivo* observational study were gathered from 25 patients. The study population was categorized while undergoing treatment for unilateral posterior edentulous space or with multiple posterior endosseous implants placed in a single quadrant, and with prosthetic rehabilitation yet to be completed in the outpatient Department of Oral Implantology. Ethical approval was obtained from the Institutional Ethics Committee (IRB no. (ETHICS/ABSMIDS/346/2023), and all participants provided written informed consent.

## Criteria for selection

### Inclusion criteria

Patients with a unilateral posterior edentulous space or multiple posterior endosseous implants placed in a single quadrant with prosthetic rehabilitation yet to be completed were recruited for the study. Only cases in which the antagonist was natural dentition were included. Only those with good oral hygiene and sound occlusion on the contralateral side were included. Participants were required to be over 20 years of age, willing to comply with oral health care instructions and follow-up visits, and to provide informed consent for documentation and public presentation of their clinical data.

### Exclusion criteria

Patients with a history of parafunctional habits such as bruxism, those experiencing temporomandibular joint pain or receiving occlusal adjustment therapy after prosthesis insertion, individuals with poor oral hygiene, and patients diagnosed with generalized periodontitis were excluded from the study.

This study was designed as a split-mouth, observational, prospective follow-up investigation. Sample size estimation was performed using OpenEpi software, with a 95% confidence level, 80% power, a 1:1 allocation ratio, and a 5% alpha error. The mean and standard deviation were derived from Luo et al. (2), resulting in a final sample size of 25 subjects.

The contralateral natural dentition was selected as an internal control to minimize interindividual variability in occlusal force patterns, neuromuscular dynamics, and masticatory function. Because occlusal load distribution varies significantly between individuals, intra-arch comparison within the same patient provides a more reliable baseline for evaluating functional adaptation of implant-supported prostheses over time.

**Figure 1:**
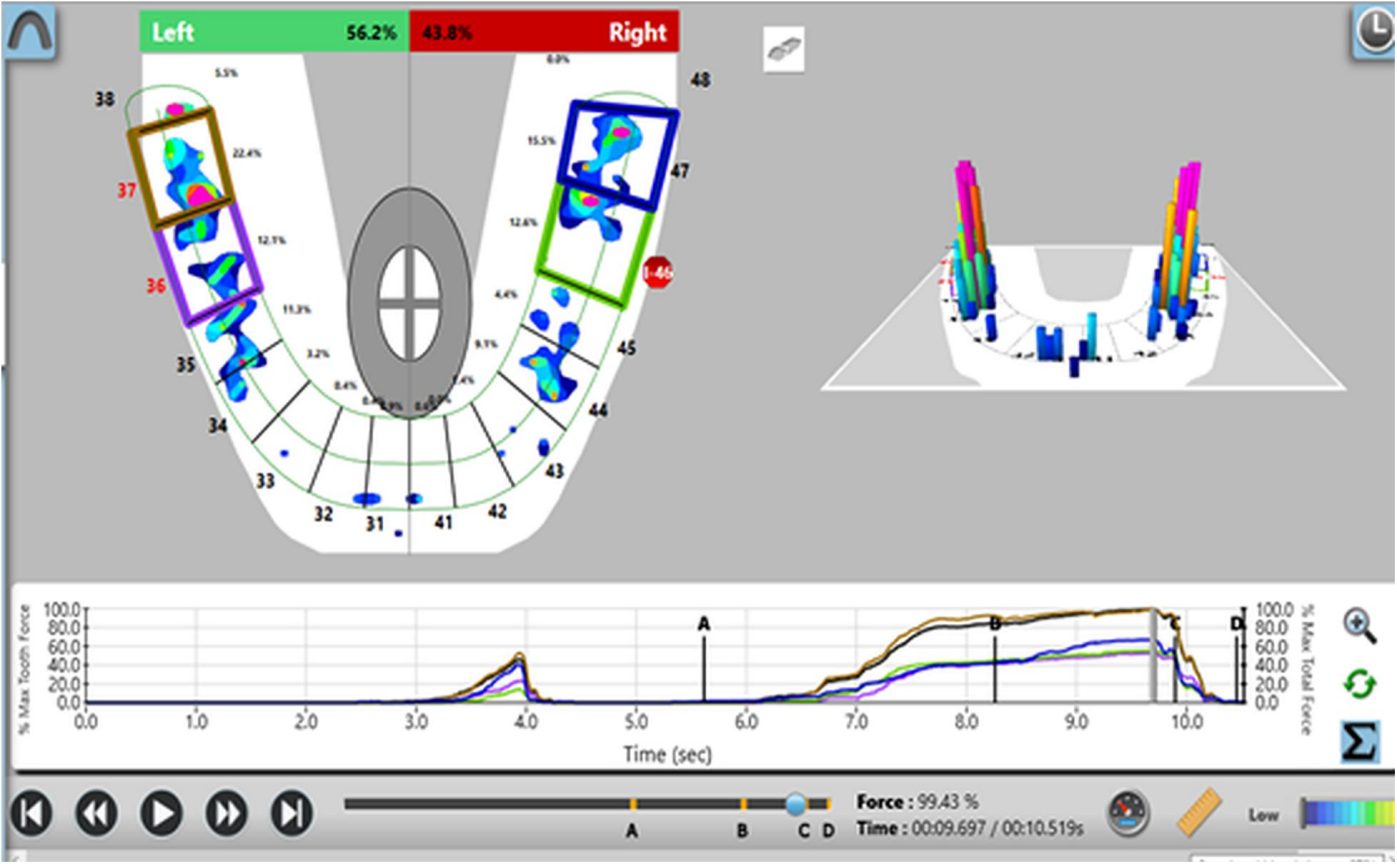
Analysis of force distribution and time parameter before prosthetic rehabilitation of implant with respect to first molar and second molar, left (36,37).

**Figure 2:**
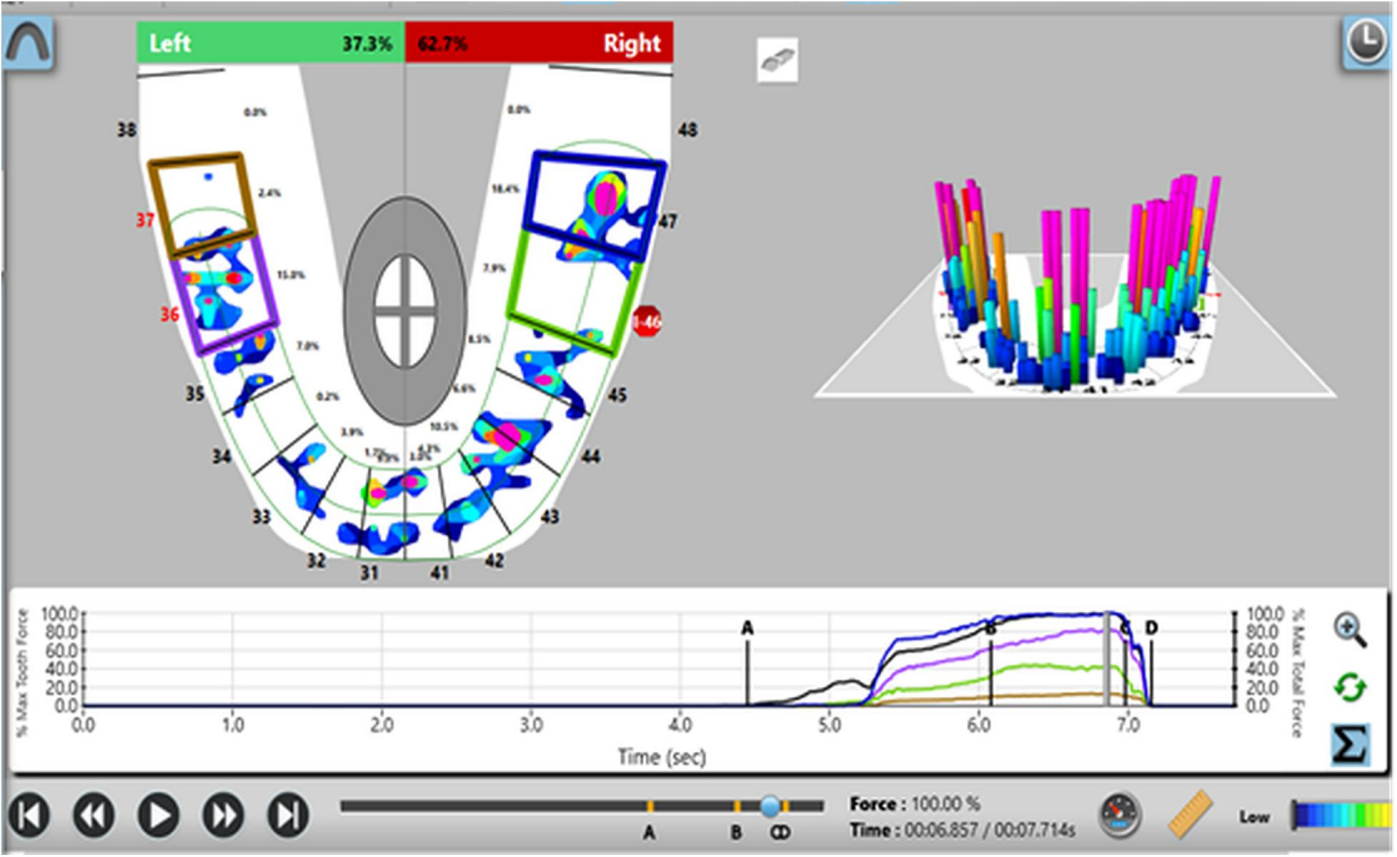
Analysis of force distribution and time parameter after prosthetic rehabilitation of implant with respect to first molar and second molar, left (36,37).

**Figure 3:**
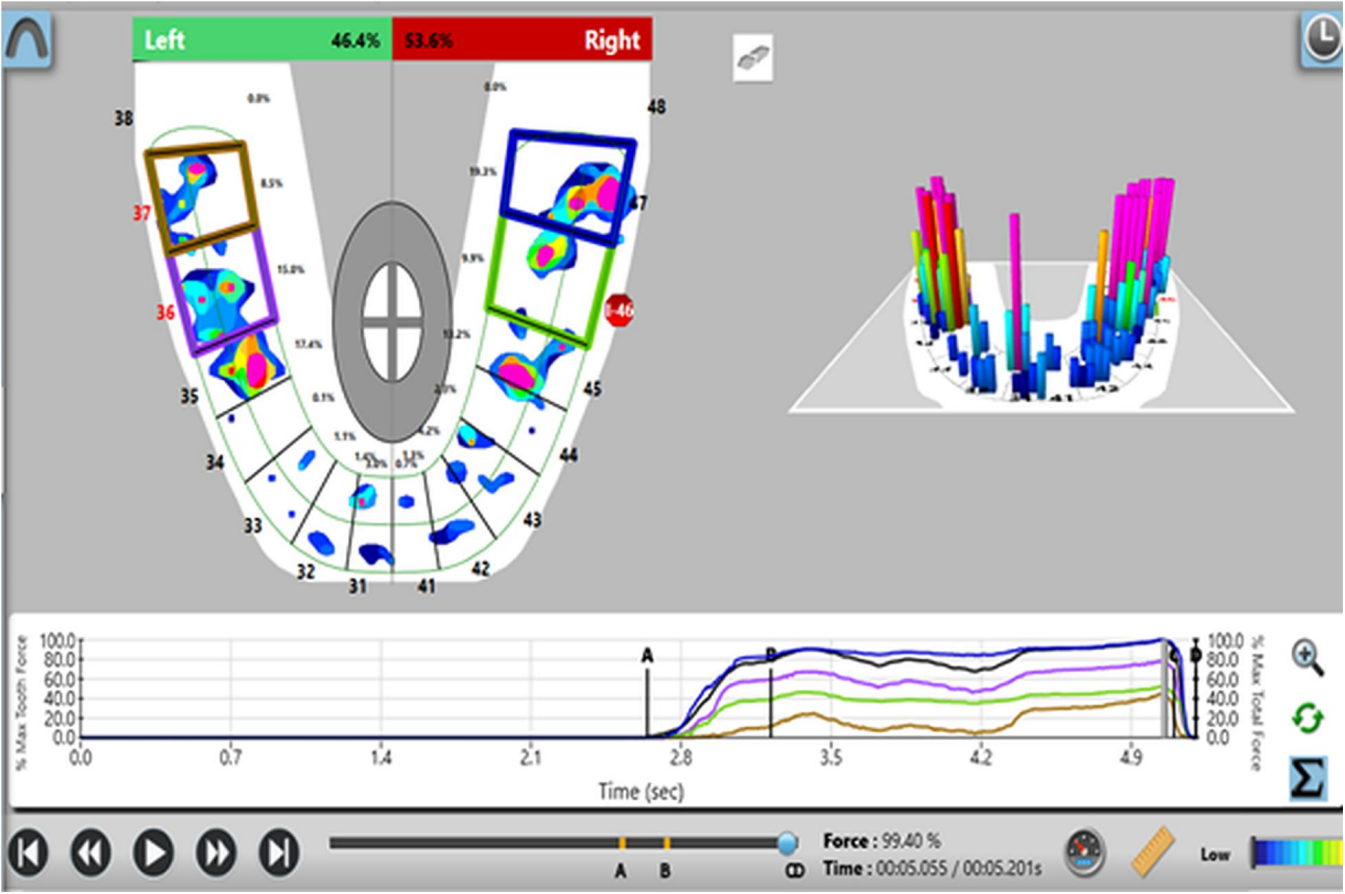
Analysis of force distribution and time parameter at 3 month follow up.

**Figure 4:**
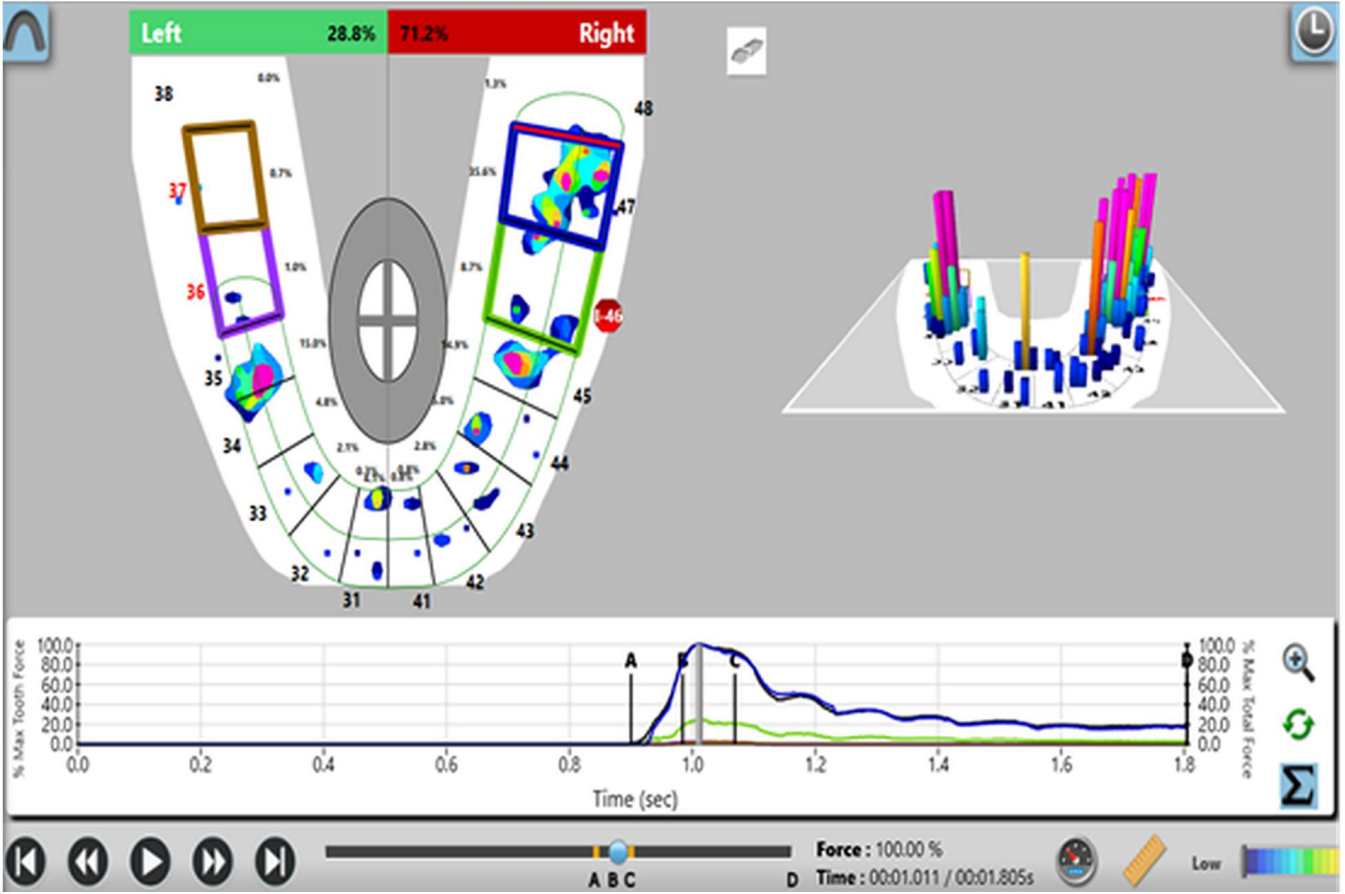
Analysis of force distribution and time parameter at 6 month follow up.

Participants were screened and selected based on predefined clinical criteria. Relative occlusal force percentages were measured using the T-Scan III system (Tekscan Inc.) with the Novus 9.0.1 software and corresponding sensor at maximal intercuspation. Baseline occlusal force data were recorded for control teeth prior to prosthesis placement (Figure 1). After prosthesis insertion, occlusal adjustments were performed using 30 µm articulating paper at maximum bite force. The relative force exerted by the implant-supported prosthesis was then compared to the corresponding contralateral natural tooth using the T scan system (Figure 2). This procedure was repeated at 3-month and 6-month follow-up visits (Figures 3, 4).

Occlusion Time (OT) was defined as the duration from initial tooth contact to maximum intercuspation. Tooth Occlusion Time (TOT) referred specifically to this interval for the control tooth, while Implant Occlusion Time (IOT) denoted the interval for the implant prosthesis. IOT reflects the duration of implant involvement in occlusal contact and may be influenced by patient-specific and operator-dependent factors. The IOT-to-OT ratio was employed as a functional indicator of occlusal contact timing for implant-supported prostheses (2). Similar ratio was employed for assessment of occlusion time for the control tooth, i.e., TOT-to-OT to eliminate discrepancy.

Data were entered into Microsoft Excel and analyzed using SPSS software (version 26.0; IBM Corp., Armonk, NY, USA). The normality of distribution for continuous variables was assessed using the Shapiro–Wilk test. Since the data did not demonstrate normal distribution, non-parametric statistical tests were employed.

Given the split-mouth design of the study, paired analyses were performed. Intergroup comparisons between the implant-supported prosthesis side (experimental group) and the contralateral natural dentition side (control group) at each time interval (baseline, immediate, 3 months, and 6 months) were carried out using the **Wilcoxon signed-rank test**.

For within-group comparisons across the four time intervals, the **Friedman test** was applied to evaluate changes over time. When multiple pairwise comparisons were performed, a **Bonferroni correction** was applied to control for inflation of the family-wise error rate. Accordingly, the adjusted level of statistical significance was set at *P* < 0.0125 for pairwise comparisons.

Categorical variables were analyzed using appropriate non-parametric methods. All results are presented as median and interquartile range (IQR) for continuous variables and as frequencies and percentages for categorical variables. A *p*-value less than 0.05 was considered statistically significant unless otherwise adjusted for multiple comparisons.

## Results

**Figure 5:**
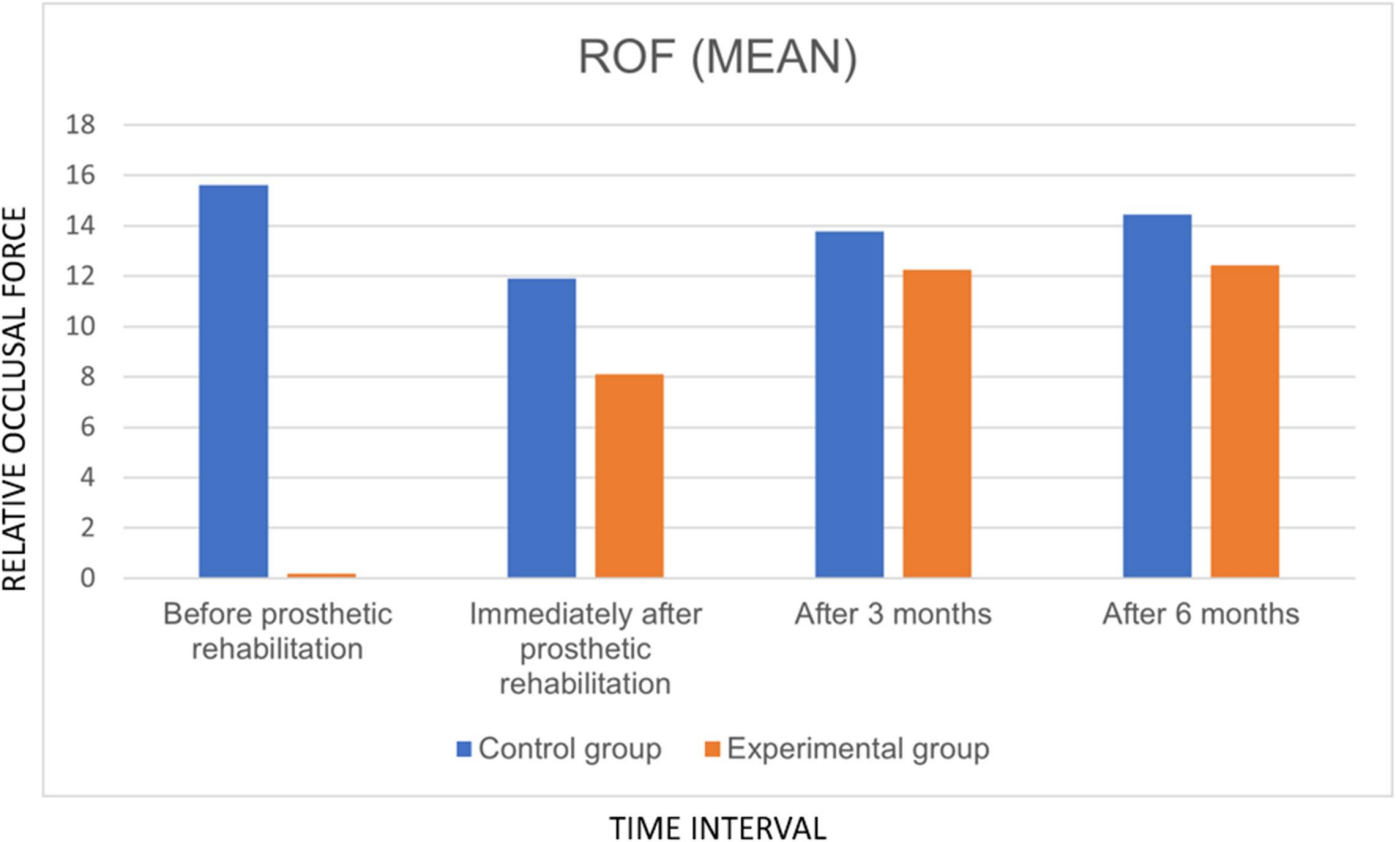
Graph showing comparison of mean of relative occlusal force between implant supported prosthesis and the control teeth at various time intervals.

The mean relative occlusal force between control and experimental groups at various time points was analyzed using the Wilcoxon signed-rank test (Tables 1, 2). Statistically significant differences were observed at all intervals except at the 3-month follow-up (*P* < 0.05). A marked disparity in occlusal force was noted before prosthetic rehabilitation, which progressively decreased over time, narrowing to a mean difference of 2.03 by the 6-month evaluation (Figure 5).

**Table 1:** Comparison of mean relative occlusal force between control and experimental groups at different time intervals.

ROF (mean)	Groups	N	Mean	Std. Deviation	*P*-value
Before prosthetic rehabilitation	Control	25	15.622	7.0076	0.000*
	Experimental	25	.190	.2969	
Immediately after prosthetic rehabilitation	Control	25	11.892	4.2667	0.003*
	Experimental	25	8.100	4.3982	
After 3 months	Control	25	13.776	4.0554	0.221
	Experimental	25	12.260	3.9176	
After 6 months	Control	25	14.462	3.8768	0.040*
	Experimental	25	12.432	3.6590	

ROF, Relative occlusal force.

**Table 2:** Comparison of mean occlusal contact time between groups at varying time intervals.

TOT/OT	Groups	N	Mean	Std. Deviation	*P*-value
Before treatment	Control	25	.3840	.10886	0.000*
	Experimental	25	.1588	.05874	
Immediately after treatment	Control	25	.4314	.10825	0.000*
	Experimental	25	.3338	.10933	
After 3 months	Control	25	.5002	.09848	0.000*
	Experimental	25	.4176	.10402	
After 6 months	Control	25	.5606	.08488	0.004*
	Experimental	25	.5152	.10180	

TOT, Tooth occlusion time; OT, Occlusion time.

Within-group comparisons across different time intervals were analyzed using the Friedman test. A statistically significant change in relative occlusal force was observed over time for the implant-supported prosthesis (*P* < 0.001), indicating progressive equilibration of occlusal forces during the follow-up period. Similarly, occlusion time demonstrated a significant temporal variation (*P* < 0.001), reflecting gradual adaptation toward the occlusal pattern of natural dentition.

**Figure 6:**
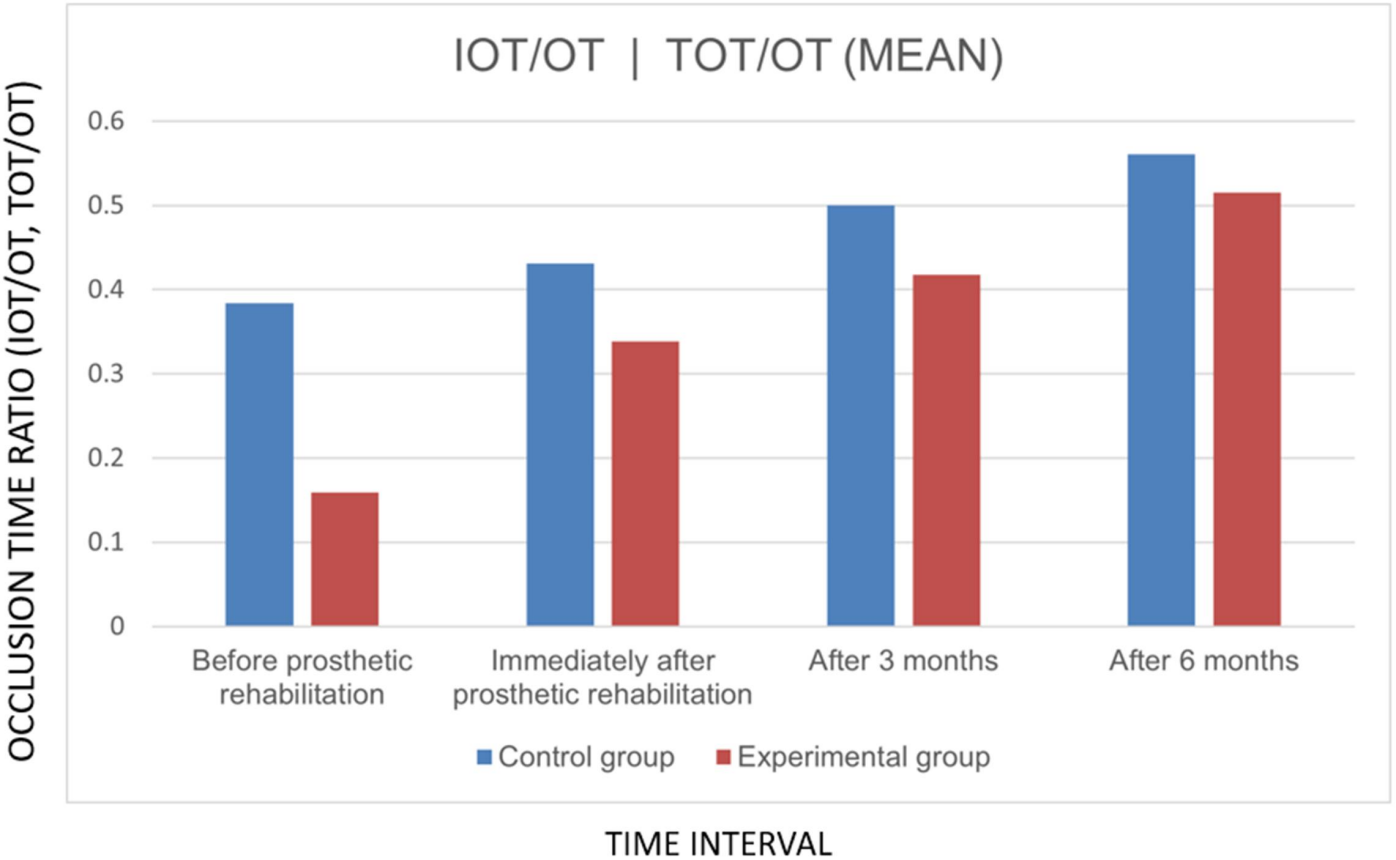
Graph showing comparison of mean of IOT/OT and TOT/OT at various time intervals.

Similarly, comparison of mean occlusal contact times between groups across different time intervals revealed statistically significant differences at all four time points (*P* <  0.05). By the end of the study, the control group demonstrated a slightly higher mean occlusion time (0.5606 ± 0.08) compared to the experimental group (0.5152 ± 0.10) (Figure 6).

A comparison between the anterior and posterior segments of the implant-supported prosthesis showed no statistically significant difference pre-rehabilitation or at the 3-month follow-up (*P* > 0.05p). However, a highly significant difference was noted immediately post-treatment and at the 6-month evaluation (*P* < 0.001). At the 3-month mark, the posterior segment exhibited a higher mean occlusal force value of 5.675. By contrast, at the 6-month follow-up, the anterior segment demonstrated an increased mean value of 4.922, compared to 3.882 for the posterior segment of the implant-supported prosthesis (Table 3).

**Table 3:** Comparison of relative occlusal force between anterior and posterior segment of prosthesis in the experimental group—Wilcoxon signed rank test.

Experimental group (ROF)	Groups	N	Mean	Std. Deviation	*P*-value
Before prosthetic rehabilitation	Anterior segment of prosthesis	25	.204	.3155	0.449
	Posterior segment of the prosthesis	25	.176	.3745	
Immediately after prosthetic rehabilitation	Anterior segment of the prosthesis	25	11.076	6.0533	0.000*
	Posterior segment of the prosthesis	25	5.124	3.4718	
After 3 months	Anterior segment of the prosthesis	25	11.208	3.5899	0.069
	Posterior segment of the prosthesis	25	13.312	5.6750	
After 6 months	Anterior segment of the prosthesis	25	14.236	4.9222	0.000*
	Posterior segment of the prosthesis	25	12.628	3.8816	

ROF, Relative occlusal force.

The mean occlusal contact time between the anterior and posterior segments of the implant-supported prosthesis in the experimental group showed a statistically significant difference at the 3-month follow-up (*P* < 0.05), whereas no significant differences were noted at other time points (*P* > 0.05). At 3 months, the anterior segment demonstrated a higher mean occlusal contact time (0.437) compared to the posterior segment (0.398) (Table 4).

**Table 4:** Comparison of mean occlusal contact time of anterior and posterior segment of prosthesis in the experimental group.

Experimental group (IOT/OT)	Groups	*N*	Mean	Std. Deviation	*P*-value
Before prosthetic rehabilitation	Anterior segment of prosthesis	25	.00	.000	1.000
	Posterior segment of the prosthesis	25	.00	.000	
Immediately after prosthetic rehabilitation	Anterior segment of the prosthesis	25	.3500	.12055	0.144
	Posterior segment of the prosthesis	25	.3176	.11748	
After 3 months	Anterior segment of the prosthesis	25	.4368	.11682	0.030*
	Posterior segment of the prosthesis	25	.3984	.10495	
After 6 months	Anterior segment of the prosthesis	25	.5340	.12352	0.064
	Posterior segment of the prosthesis	25	.4964	.09721	

IOT, Implant occlusion time; OT, Occlusion time.

## Discussion

The present prospective study evaluated longitudinal changes in relative occlusal force and occlusion time in posterior multiple implant-supported fixed prostheses. The findings demonstrate that occlusal parameters are not static following prosthesis delivery but undergo progressive adaptation over time.

Immediately after restoration, relative occlusal force was significantly lower on implant-supported prostheses compared to contralateral natural dentition. This observation reflects the clinical application of implant-protected occlusion principles, wherein implant contacts are intentionally light or slightly delayed to reduce overload risk. Similar findings were reported by Madani et al. (8), who observed lower force values on posterior implant restorations compared to natural teeth.

Interestingly, force concentration was initially greater in the anterior segment of the prosthesis immediately post-rehabilitation. This phenomenon may be explained by neuromuscular physiology at maximum intercuspation. Elevator muscle activity, modulated by periodontal mechanoreceptors, plays a critical role in fine-tuning occlusal contacts (9, 10). In partially edentulous segments, reduced proprioceptive feedback may alter mandibular posture and occlusal contact distribution. Intentional infraocclusion of implant prostheses at delivery may further contribute to transient anterior force predominance.

Longitudinal analysis revealed a progressive posterior shift and equilibration of occlusal forces. By six months, anterior–posterior force discrepancies had narrowed considerably, suggesting adaptive redistribution within the arch. These findings parallel observations by Roque et al. (12), who reported occlusal pressure redistribution in single implant restorations opposing natural dentition. The results reinforce the concept of arch-wide functional interdependence, wherein occlusal changes in one segment influence load distribution across adjacent and opposing units.

Occlusion time analysis further supported dynamic adaptation. Although implant contacts were initially lighter and slightly shorter in duration, occlusion time progressively increased over the follow-up period, approaching values observed in natural dentition. This trend aligns with reports by Luo et al. (2), who documented significant increases in implant occlusion time over extended follow-up periods.

Beyond eruptive adaptation of antagonistic teeth, neuromuscular coordination within the stomatognathic system likely contributes to these changes. Continuous eruption of natural teeth, minor occlusal wear, and muscle-mediated functional adjustments collectively influence contact timing and force intensity. Because implants lack periodontal proprioception and physiologic mobility, they rely on surrounding arch adaptation for equilibration. This biomechanical disparity explains why occlusal reassessment remains critical even when IPO principles are followed at insertion.

Clinically, these findings emphasize that implant-supported prostheses should not be considered functionally stable immediately after delivery. Progressive equilibration occurs within the first six months, and periodic occlusal evaluation is advisable to prevent potential overload and mechanical complications.

While digital occlusal analysis provides quantitative precision, many practices may not have access to T-Scan technology. In such settings, careful use of articulating paper in conjunction with shimstock verification and bilateral simultaneous contact assessment can serve as practical alternatives. Regardless of the method used, scheduled follow-up appointments remain essential for early detection of occlusal discrepancies.

## Limitations

For the study, follow-up duration was limited to 6 months, which may not fully capture long-term occlusal adaptation. The sample size, although statistically calculated, was relatively modest. Additionally, occlusal analysis was performed under maximal intercuspation and did not include dynamic functional movements. Finally, while T-Scan quantifies occlusal force distribution, it does not directly measure stress at the bone–implant interface. Future longitudinal studies with larger cohorts and extended follow-up are warranted.

## Conclusion

Within the limitations of this 6-month prospective study:

1. Relative occlusal force and occlusion time in posterior multiple implant-supported prostheses demonstrated progressive changes over time
2. Implant-supported prostheses demonstrated gradual equilibration toward natural dentition within the dental arch.
3. Periodic occlusal reassessment is recommended to minimize the risk of biomechanical overload.

## Data Availability

The raw data supporting the conclusions of this article will be made available by the authors, without undue reservation.
